# Public health round-up

**DOI:** 10.2471/BLT.19.011019

**Published:** 2019-10-01

**Bahamas recovering after Hurricane Dorian**

The remains of a clapboard house in New Plymouth, Green Turtle Cay, a barrier island off Great Abaco in the northern Bahamas. Home to some 500 people, Green Turtle Cay was devastated by Hurricane Dorian at the beginning of September.

**Figure:**
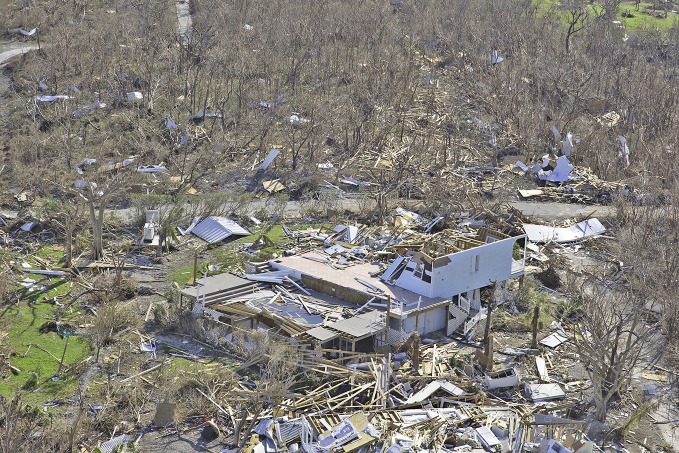
PAHO/Victor Ariscain

## Hurricane Dorian response

The Pan American Health Organization/World Health Organization (PAHO/WHO) deployed emergency teams in response to Hurricane Dorian, which made landfall in Elbow Cay, Bahama, on 1 September.

PAHO/WHO had already deployed rapid response team experts to the Bahamas ahead of the hurricane to support health authorities and humanitarian response efforts. As of 8 September, 14 PAHO experts had been sent to the disaster zone to provide logistics, civil and military coordination, information management, epidemiological surveillance, communications, and coordination.

As of 11 September, 50 deaths had been officially reported, but more were expected. It was estimated that more than 76 000 people had been affected, hundreds seeking shelter in the disaster zone. The health system suffered significant destruction of equipment and medical supplies in Abaco and Grand Bahama.

PAHO/WHO called for an initial US $3.5 million from donors to cover short- term healthcare, water and sanitation, epidemiological surveillance and vector-control needs in the islands most affected for the next six months.

https://www.paho.org/disasters/?lang=en

## More than 2000 deaths in Ebola outbreak

By 7 September, 2064 people had died in the Democratic Republic of the Congo’s ongoing Ebola virus outbreak, roughly two thirds of the 3079 infected as of that date. The outbreak began in August 2018.

Continued unrest and community reticence to cooperate with health workers in some parts are hampering epidemic response efforts, preventing the timely detection and isolation of people infected with the virus, and thus favouring continued transmission.

Issues of concern to health authorities and supporting agencies include the high proportion of Ebola deaths still occurring in the community, the relatively low proportion of people infected who were previously identified as contacts and under surveillance, and transmission linked to possible nosocomial infection.

At the beginning of September, WHO called for partners to follow through on commitments made and to ensure adequate funding going forward. As of 4 September, funding covered financing needs until the end of the month. Further resources are needed to fund the response through to December 2019.

https://www.who.int/csr/don/06-september-2019-ebola-drc/en/

## Sudan cholera response

Between 28 August and 10 September, the Republic of Sudan’s Federal Ministry of Health reported at least 51 cases of acute watery diarrhoea. Four of 6 samples sent to the ministry's National Public Health Laboratory tested positive for *Vibrio cholerae*. 

WHO sent a team of public health experts to Blue Nile State, where the cases were reported. The WHO team is working with health authorities to strengthen disease surveillance, provide medical treatment, distribute laboratory supplies, monitor water quality and chlorinate public water supplies, and promote health education and hygiene among affected and at-risk communities. As of 11 September, two cholera treatment centres were treating patients in Blue Nile State, and a dedicated isolation centre had been established for cholera case management. 

http://www.emro.who.int/sdn/sudan-news/who-responds-to-cholera-cases-in-sudan.html

## WHO ships supplies to Libya

At the beginning of September, WHO delivered medicines and medical supplies to Libya to support the country’s health system which has been overwhelmed as a result of the ongoing conflict there. The supplies, sufficient to treat 220 000 patients for 3 months, were shipped to primary health care centres, field hospitals and referral hospitals. 

http://www.emro.who.int/lby/libya-news/who-sends-medical-supplies-across-libya.html

## Genome editing registry launched

A WHO expert advisory committee approved the first phase of a new global registry to track research on human genome editing. Supported by WHO’s International Clinical Trials Registry Platform, the first phase of the project will include somatic and germline clinical trials.

At their 28-29 August meeting, the committee called on all researchers involved in relevant research and development to register their trials and announced that it will be undertaking online consultation regarding genome editing governance.

Addressing the committee, WHO Director-General Tedros Adhanom Ghebreyesus emphasized that countries should halt further work on human germline genome editing in human clinical applications until the technical and ethical implications have been properly considered.

https://www.who.int/news-room/detail/29-08-2019-who-launches-global-registry-on-human-genome-editing

## Measles surges in European Region

Approximately 90 000 measles cases were reported in the WHO European Region in the first half of 2019, surpassing the 84 462 reported for the whole of 2018.

Following several years of steady progress toward elimination in the region, there has been a decline in the number of countries judged to have achieved or sustained elimination of the disease. Four countries (Albania, Czechia, Greece and the United Kingdom of Great Britain and Northern Ireland) lost their measles elimination status.

http://www.euro.who.int/en/media-centre/sections/press-releases/2019/european-region-loses-ground-in-effort-to-eliminate-measles

## Revised guidance on contraceptive use

WHO revised its guidance on contraceptive use to reflect new evidence that women at high risk of HIV infection can use any form of reversible contraception, including progestogen-only injectables, implants and intrauterine devices.

Because these contraceptive methods do not protect against HIV and other sexually transmitted infections, the guideline, *Medical eligibility criteria for contraceptive use*, emphasizes the importance of condom use where there is a risk of such infections. WHO also recommends considering offering pre-exposure prophylaxis in settings where the incidence of HIV is above 3%.

www.who.int/reproductivehealth/publications/contraceptive-eligibility-women-at-high-risk-of-HIV/en/

## WHO backs open access

WHO became the first United Nations agency to join cOAlition S, a coalition of research funders and charitable foundations committed to ensuring open access to research publications.

cOAlition S is built around Plan S, which comprises 10 principles to ensure that results from publicly-funded research is published in open access journals, on open access platforms, or made immediately available through open access repositories.

“By joining this coalition, we believe we can accelerate progress towards universal free access to health research,” said WHO Chief Scientist Soumya Swaminathan at the 29 August launch event.

https://www.who.int/news-room/detail/29-08-2019-who-joins-coalition-for-free-digital-access-to-health-research

**Cover photo**

A teacher and a student at the National School for Deaf Children in Kabul, Afghanistan.

**Figure:**
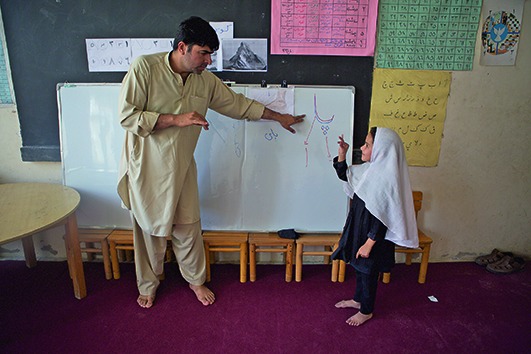
Rada Akbar.

## Fighting vaccine misinformation

Facebook has committed to direct millions of its users to WHO-published vaccine information to ensure that reliable, evidence-based health messages reach people who need them.

On September 4, WHO announced that it had been in discussions with Facebook for several months regarding ways to address the issue of vaccine misinformation and welcomed Facebook’s commitment to ensuring that users can find facts about vaccines across Instagram, Facebook Search, Facebook Groups, Facebook Pages and Facebook Forums where people seek out information and advice.

https://www.who.int/news-room/detail/04-09-2019-vaccine-misinformation-statement-by-who-director-general-on-facebook-and-instagram

## Suicide prevention

WHO co-launched a suicide prevention campaign on 10 September. Titled *40 seconds of action* to reflect the fact that every 40 seconds someone on the planet takes his or her own life. The campaign was launched in collaboration with the World Federation for Mental Health, the International Association for Suicide Prevention and United for Global Mental Health. The campaign will culminate on World Mental Health Day on 10 October.

To coincide with the launch of the suicide prevention campaign, WHO published *Preventing suicide: a resource for pesticide registrars and regulators*, which reflects a growing body of evidence indicating that regulations to prohibit the use of certain pesticides can lead to reductions in national suicide rates.

https://www.who.int/news-room/detail/09-09-2019-suicide-one-person-dies-every-40-seconds

**Looking ahead**

8–10 October - World Conference on Drowning Prevention. Durban, South Africa.

22–24 October - Fifth High-level Meeting on Transport, Health and Environment. Vienna, Austria.

27–29 October - Global Health Summit. Berlin, Germany.

